# Sex-Dependent Influences of Obesity on Cerebral White Matter Investigated by Diffusion-Tensor Imaging

**DOI:** 10.1371/journal.pone.0018544

**Published:** 2011-04-11

**Authors:** Karsten Mueller, Alfred Anwander, Harald E. Möller, Annette Horstmann, Jöran Lepsien, Franziska Busse, Siawoosh Mohammadi, Matthias L. Schroeter, Michael Stumvoll, Arno Villringer, Burkhard Pleger

**Affiliations:** 1 Max Planck Institute for Human Cognitive and Brain Sciences, Leipzig, Germany; 2 Wellcome Trust Centre for Neuroimaging, UCL Institute of Neurology, University College London, London, United Kingdom; 3 Department of Medicine, University Hospital Leipzig, Germany; 4 Integrated Research and Treatment Center (IFB) Adiposity Diseases, Leipzig, Germany; 5 Clinic for Cognitive Neurology, University Hospital Leipzig, Leipzig, Germany; Brigham & Women's Hospital, and Harvard Medical School, United States of America

## Abstract

Several studies have shown that obesity is associated with changes in human brain function and structure. Since women are more susceptible to obesity than men, it seems plausible that neural correlates may also be different. However, this has not been demonstrated so far. To address this issue, we systematically investigated the brain's white matter (WM) structure in 23 lean to obese women (mean age 25.5 y, std 5.1 y; mean body mass index (BMI) 29.5 kg/m^2^, std 7.3 kg/m^2^) and 26 lean to obese men (mean age 27.1 y, std 5.0 y; mean BMI 28.8 kg/m^2^, std 6.8 kg/m^2^) with diffusion-weighted magnetic resonance imaging (MRI). There was no significant age (*p*>0.2) or BMI (*p*>0.7) difference between female and male participants. Using tract-based spatial statistics, we correlated several diffusion parameters including the apparent diffusion coefficient, fractional anisotropy (FA), as well as axial (λ_∥_) and radial diffusivity (λ_⊥_) with BMI and serum leptin levels. In female and male subjects, the putative axon marker λ_∥_ was consistently reduced throughout the corpus callosum, particularly in the splenium (*r* = −0.62, *p*<0.005). This suggests that obesity may be associated with axonal degeneration. Only in women, the putative myelin marker λ_⊥_ significantly increased with increasing BMI (*r* = 0.57, *p*<0.005) and serum leptin levels (*r* = 0.62, *p*<0.005) predominantly in the genu of the corpus callosum, suggesting additional myelin degeneration. Comparable structural changes were reported for the aging brain, which may point to accelerated aging of WM structure in obese subjects. In conclusion, we demonstrate structural WM changes related to an elevated body weight, but with differences between men and women. Future studies on obesity-related functional and structural brain changes should therefore account for sex-related differences.

## Introduction

Obesity develops as a consequence of an imbalance between food intake and energy consumption, resulting in a major increase of body fat. People are defined as obese when their body mass index (BMI) equals or exceeds 30 kg/m^2^. Obesity predisposes an individual to many serious diseases, such as cardio- and neurovascular diseases, diabetes mellitus, osteoarthritis [1], and various types of cancer [2]. Obesity decreases life expectancy and quality [3] and thus represents one of the most serious medical conditions worldwide and is one of the leading preventable causes of death [4]. Being overweight is often socially stigmatized and accompanied by discrimination [5]. Moreover, it is associated with severe neuropsychiatric disorders, such as depression [1] and dementia [6], [7], suggesting a bodyweight-dependent influence on brain structure and function. Weight increase is also observed as an effect of treatment for various disorders of the central nervous system using deep brain stimulation [8].

Several studies suggest that being overweight predisposes individuals to smaller brain volume [9], [10], brain atrophy [11], or reduced gray matter (GM) density [12]. Using computed tomography (CT), Gustafson et al. [11] showed brain atrophy in the temporal lobe related to a higher BMI, suggesting that the risk of brain atrophy is increased with higher BMI. Other studies used structural magnetic resonance imaging (MRI) with voxel- and tensor-based methods in order to provide a more specific localization of structural brain changes. Using a set of *T*_1_-weighted (*T*_1_w) images, Pannacciulli et al. [12], [13] showed an inverse correlation between GM density and BMI in brain areas involved in the regulation of taste, reward, and behavioral control. Walther et al. [14] also found an inverse relationship between BMI and GM density in several brain regions, particularly in the orbito-frontal cortex—a key region for the representation of flavor and rewarding properties of food [15]. Together, these findings suggest structural changes in reward or reward-associated brain regions, probably rendering obesity as a form of addiction.

Given widespread structural changes in GM, it seems plausible that white matter (WM) is also affected, and a few studies have suggested this to be the case: Analyzing *T*_1_w images with tensor-based morphometry, Raji et al. [16] found atrophy in the corona radiata in overweight subjects. Haltia et al. [17] also studied WM on *T*_1_w images, and reported WM changes as an effect of dieting.

Another MRI-based approach for investigating WM structure is diffusion tensor imaging (DTI) [18], [19]. From the diffusion tensor, several parameters which are related to different aspects of underlying WM structure, including fiber orientation, can be derived. A measure of the mean diffusivity in a voxel is the apparent diffusion coefficient (ADC). Another frequently used measure is the fractional anisotropy (FA) [20], which describes the degree of anisotropy of the diffusion process in the tissue. Fractional anisotropy is discussed as reflecting WM fiber density, parallelism of axons, axonal diameter, and myelination, and a reduced FA is often interpreted as showing a decline in WM “integrity” [21]. For example, several studies showed reduced FA in diseases such as hypoxic-ischemic encephalopathy [22], traumatic brain injury [23], multiple sclerosis [24], attention-deficit hyperactivity disorder [25], epilepsy [26], schizophrenia [27], bipolar disorder [28], depression [29], [30], types of dementia such as Alzheimer's disease [7], [31] and Parkinson's disease [32]. This indicates that the FA is a sensitive but rather unspecific marker of structural changes. Further parameters, which can be derived from the diffusion tensor are the axial and radial diffusivity, λ_∥_ and λ_⊥_, respectively. They provide measures for diffusivity along and perpendicular to the fiber tract within a voxel, respectively, and have been suggested for differentiation between axonal and myelin degeneration [33].

Few studies so far have used diffusion imaging in obesity. Alkan et al. [34] used the ADC to compare the diffusion of water molecules within cerebral tissue between obese and lean subjects. They showed a correlation between BMI and ADC in various brain regions. Investigating the obesity risk in healthy older adults, Marks et al. [35] showed significant correlations between BMI and FA. A limitation of this study, however, was the small sample size (15 subjects), which did not allow an investigation of sex-dependent effects. Since men and women show differences in food selection and intake, and women are more susceptible to obesity than men [36], the investigation of sex-dependent differences in brain structure and function seems promising for a better understanding of obesity-related cerebral mechanisms.

BMI is the most widely used parameter to identify weight problems within a population. It is a heuristic measure of healthy body weight based on the individual's weight and height assuming an average body composition. However, overweight and obese individuals are generally defined as having more body fat than average and optimally healthy individuals. To account for excess body fat, we collected blood samples from most of our subjects to determine serum levels of leptin, which reflects the percentage of body fat with a high degree of accuracy [37]. Leptin is one of the most important adipose-derived hormones, which signals information about long-term energy stores to the brain and modulates its reward function [38], [39]. It plays a key role in regulating energy intake and energy expenditure, including appetite and metabolism. Since its blood levels are directly correlated with its central nervous concentrations, leptin represents an appropriate marker for the influence of body adiposity on brain function and structure [40]. Few studies have so far used leptin to show related structural brain changes in *T*_1_w imaging [13], [41].

Thus, in the current study, we investigated sex-dependent correlations between the diffusion parameters ADC, FA, λ_∥_, and λ_⊥_, and the obesity parameters BMI and blood concentrations of leptin.

## Materials and Methods

### Participants

Forty-nine volunteers (23 women; mean age 26.4 y, std 5.0 y; mean BMI 29.1 kg/m^2^, std 7.0 kg/m^2^) participated in the experiment. There were no significant differences in age (women: mean age 25.5 y, std 5.1 y; men: mean age 27.1 y, std 5.0 y; *p*>0.2) or BMI (women: mean BMI 29.5 kg/m^2^, std 7.3 kg/m^2^; men: mean BMI 28.8 kg/m^2^, std 6.8 kg/m^2^; *p*>0.7) between women and men.

Serum leptin concentrations were available for a subsample of 44 subjects (22 women; mean age 26.1 y, std 5.1 y; mean BMI 29.2 kg/m^2^, std 7.2 kg/m^2^). In this subgroup, there was again no significant age (women: mean age 25.7 y, std 5.1 y; men: mean age 26.6 y, std 5.2 y) or BMI (women: mean BMI 29.4 kg/m^2^, std 7.4 kg/m^2^; men: mean BMI 29.0 kg/m^2^, std 7.1 kg/m^2^) difference between women and men (*p*>0.5). For further details, see Table S1 in the supplementary material. Exclusion criteria were a history of neuropsychiatric diseases, smoking, diabetes mellitus, known contraindications to MRI, and abnormalities on a *T*_1_w MRI scan. The study was conducted in accordance with the Declaration of Helsinki and was approved by the Ethics Committee of the Faculty of Medicine of the Leipzig University. Subjects gave their informed written consent before participating in the study.

Directly before the MRI session, body size and body weight were measured using digital scales. Blood from a peripheral venous puncture in the elbow flexure was withdrawn into a serum vacutainer and centrifuged at 4°C for 10 minutes at a relative centrifugal force of 3500 *g* to separate the serum, which was stored at −80°C. Finally, the serum leptin concentrations were determined by enzyme-linked immunosorbent assays (ELISA, Mediagnost, Reutlingen, Germany). The amount of body fat was determined by bioelectrical impedance analysis (BIA) using electrodes on the participant's hand and foot (Data-Input GmbH, Darmstadt, Germany). For computing the fat-to-lean mass ratio (FLMR), the lean mass was obtained from the difference between body mass and body fat. Fat-to-lean mass ratio data were available for 20 female and 20 male participants.

In addition to the physiological parameters, we evaluated the severity of depression using the Beck Depression Inventory (BDI) measuring both the affective and somatic component. The BDI scores were also available for 20 female and 20 male participants. Correlation analyses were performed to investigate the dependence between BDI scores and obesity measures.

### Image Acquisition

Magnetic resonance imaging was performed using a 3-T TIM Trio scanner (Siemens, Erlangen, Germany) and a 12-channel head array coil. Axial diffusion-weighted (DW) images were acquired with a twice-refocused spin-echo echo-planar-imaging (EPI) sequence [42] with fat suppression and a 6/8 partial Fourier acquisition scheme combined with generalized auto-calibrating partially parallel acquisitions (GRAPPA; acceleration factor 2). Acquisition parameters were: echo time (TE) 100 ms, repetition time (TR) 12 s, image matrix 128×128, field of view (FOV) 220×220 mm^2^, bandwidth 1345 Hz/pixel. A total of 60 diffusion-encoding gradient directions were investigated with a *b*-value of 1000 s/mm^2^. Additional images without diffusion weighting were acquired at the beginning and after each block of ten DW images. The interleaved measurement of 72 axial slices with 1.7 mm thickness (no gap) covered the entire brain. The whole DW imaging series was repeated three times for subsequent signal averaging (offline), resulting in an acquisition time of approximately 45 min.

Prior to DW MRI, *T*_1_w images were acquired using a three-dimensional magnetization-prepared rapid gradient echo (MP-RAGE) sequence (sagittal orientation) with selective water excitation and linear phase encoding. Imaging parameters were inversion time (TI) 650 ms, TR 1.3 s, repetition time of the gradient-echo kernel 10 ms, TE 3.93 ms, flip angle 10°, bandwidth 130 Hz/pixel, image matrix 256×240, FOV 256×240 mm^2^, slab thickness 192 mm, 128 partitions, 95% slice resolution, 2 averages. To avoid aliasing, oversampling was performed in read direction (head to foot). Reconstructed images were obtained using zero filling with a resolution of 1×1×1 mm^3^.

### Data Analysis

The *T*_1_w scans were used for skull-stripping, and the brain images were then co-registered into Talairach space [43]. The 21 images acquired without diffusion weighting at each slice position were used to estimate motion correction parameters using rigid-body transformations [44] implemented in FSL [45]. The motion correction parameters were interpolated for the 180 DW images and combined with a global registration to the *T*_1_w anatomy computed with the same method. The gradient direction for each volume was corrected using the rotation parameters. The registered images were interpolated to the new reference frame with an isotropic voxel dimension of 1 mm, and the three corresponding acquisitions and gradient directions were averaged. Finally, a diffusion tensor was fitted to the data of each voxel and the parameters ADC, FA, λ_∥_, and λ_⊥_ were computed from the eigenvalues of the diffusion tensor. For separate analysis, the DW images of each subject were also registered to the corresponding non-DW images [46] to extract a scaling parameter along the read direction (left to right; i.e., *x*-direction), which has recently been shown to reflect effects from mechanical vibrations caused by the rapid switching of the diffusion-weighting gradients [47].

Voxel-wise statistical analyses of the diffusion parameters were carried out using Tract-Based Spatial Statistics TBSS [48] as implemented in FSL. All subjects' FA maps were first registered to a mean FA template and then skeletonized. Finally, the individual FA parameters were projected onto the template skeleton. The same transformation was applied to all other diffusion parameters. Next, the resulting data on the template skeleton were fed into voxel-wise cross-subject statistical analyses based on randomization tests [49] using 100,000 permutations. The design matrix was generated using age and BMI or leptin concentrations as covariates. Significant correlations between diffusion parameters and BMI or leptin levels were detected using threshold-free cluster enhancement and correction for multiple comparisons in order to give an inference at the *p*<0.05 (corrected) level [50]. The dependence between the diffusion parameters and body weight was investigated using both BMI and leptin levels as obesity markers. In order to investigate sex differences, all TBSS analyses were performed separately for women and men. To check the validity of the results, the correlation between FA and the obesity parameters BMI and leptin was also computed with voxel-wise parametric tests using SPM8 (http://www.fil.ion.ucl.ac.uk/spm) and an uncorrected threshold of *p*<0.001.

In addition to the voxel-wise analyses, region-of-interest (ROI) analyses were performed in the genu and splenium of the corpus callosum. Both ROIs were defined using the mean skeleton obtained with the TBSS analysis. Diffusion parameters were averaged across the ROI and correlated with BMI and leptin using partial correlation taking the age of the subjects into account. All ROI analyses were repeated using the BDI scores for partial correlation. Partial correlations and *p*-values were obtained using SPSS (http://www.spss.com) statistics.

Further analyses were performed to investigate the influence of signal-to-noise ratio (SNR) variations across subjects due to possible body-weight related changes in coil loading. For each subject, the standard deviation of the background noise was estimated using regions outside the brain in the non-DW and in the DW images. For both genu and splenium of the corpus callosum, the SNR was computed by dividing the mean signal of the respective ROI by the standard deviation of the background noise. Using permutation analyses, the correlation between FA and BMI was computed including the SNR in the genu and splenium as covariates. Additionally, the ROI analyses were performed again for both genu und splenium of the corpus callosum using the SNR in both regions as additional covariates.

## Results

### Participant Parameters

We found higher serum leptin concentrations in women than in men (women: mean 35.4 ng/ml, std 27.5 ng/ml; men: mean 8.8 ng/ml, std 13.2 ng/ml; diff: *t* = 4.09, *p*<0.001) which goes in line with recent findings [51], [52]. In both women and men, we observed a strong correlation between BMI and leptin level (women: *r* = 0.71, *p*<0.001; men: *r* = 0.94, *p*<0.001) suggesting a consistency of using BMI and serum leptin level as obesity measures. The FLMR was higher in women as compared to men (women: mean 0.54, std 0.22; men: 0.26, std 0.13; diff: *t* = 5.02, *p*<0.001) and correlated with BMI (women: *r* = 0.91, *p*<0.001; men: *r* = 0.79, *p*<0.001) and with the serum leptin level (women: *r* = 0.78, *p*<0.001; men: *r* = 0.70, *p* = 0.001). Serum leptin levels are known to be influenced by diurnal fluctuations [53] and dietary intake [52]. Although recording of leptin levels was not standardized for time of day or diet in the current study, their strong correlations with BMI and FLMR indicate that higher leptin values reflect higher amounts of body fat in both women and men.

We also investigated the relationship between depression and obesity using correlation analyses. We correlated all three BDI parameters (affective, somatic, and the sum of both components) with the obesity measures (i.e., BMI, serum leptin level, and FLMR). However, in our sample, we did not find any significant correlation between depression and obesity markers in women and men (*p*>0.05). We also found no significant differences between women and men using correlations between obesity markers and all three BDI parameters (*p*>0.05).

### Tract-Based Spatial Statistics (TBSS)

**Figure 1:**
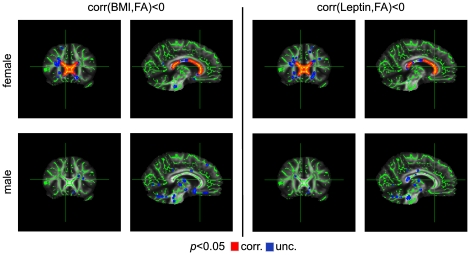
Sex-specific correlations between FA and body weight using BMI (left columns) and serum leptin level (right columns). For women (top row), we found a significant negative correlation in the entire corpus callosum (*p*<0.05, corrected). In contrast, for men (bottom row), we found no correlation even without correcting for multiple comparisons.

**Figure 2:**
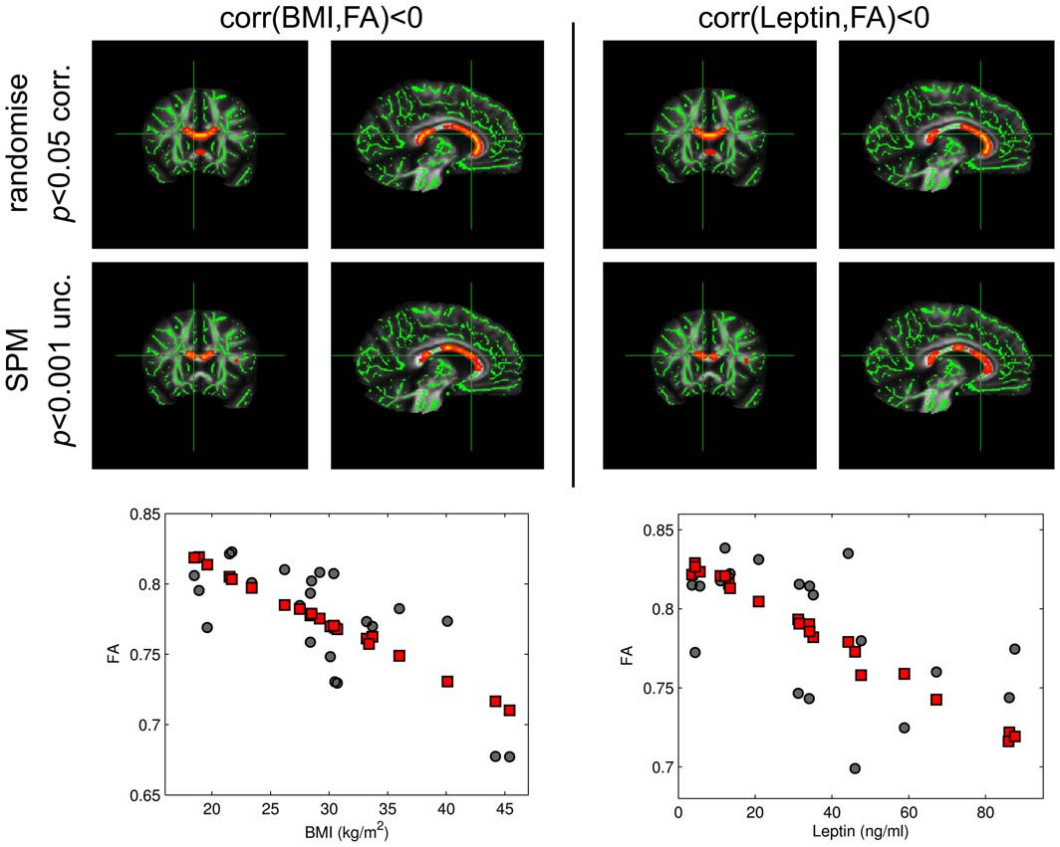
Coronal and sagittal brain slices showing a negative correlation (color-coded in red/yellow) between FA and body weight using BMI (left) and leptin levels (right) in women. The result was obtained using the ‘randomise’ tool of FSL (*p*<0.05, corrected; top row), and the statistics in SPM8 (*p*<0.001, uncorrected; bottom row). The dot plots (see bottom of the figure) show the FA values of the women in a selected voxel (green cross-hair in the brain slices). Black dots and red squares show original and age-corrected FA values, respectively.

For women, we found a significant negative correlation between FA and BMI in the entire corpus callosum (*p*<0.05, corrected; Figure 1, top row). This result was obtained with the ‘randomise’ tool of FSL (*p*<0.05, corrected) and corroborated with a parametric analysis using SPM8 (*p*<0.001, uncorrected; Figure 2). By contrast, no significant (either negative or positive) FA-BMI correlation was observed for men at *p*<0.05, even without correction for multiple comparisons (Figure 1, bottom row). In the parametric analysis, variance was also explained by the age of the subjects (see dot plots in Figure 2).

**Figure 3:**
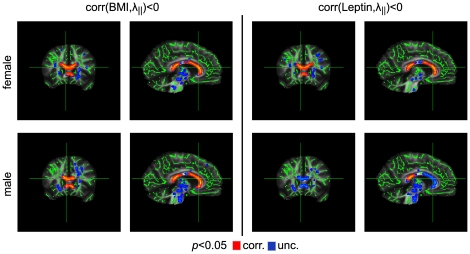
Correlation between λ_∥_ and body weight using BMI (left) and leptin levels (right). For both women and men, we found a significant negative correlation between BMI and λ_∥_ in all regions of the corpus callosum (both left columns). This result was again obtained by using the leptin level instead of BMI as an obesity marker (see right columns).

A significant negative correlation between axial diffusivity (λ_∥_) and BMI was observed in all regions of the corpus callosum for both women and men (*p*<0.05, corrected; Figure 3).

**Figure 4:**
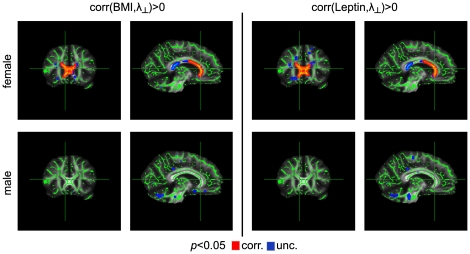
Correlation between λ_⊥_ and body weight using BMI (left) and leptin levels (right). For women, we found a significant positive correlation between BMI and λ_⊥_ in the corpus callosum (top row). For men, we found no dependency between BMI and radial diffusivity even without correction for multiple comparisons (bottom row). The same results were obtained for the leptin level instead of BMI (see right columns).

The dependence between radial diffusivity (λ_⊥_) and body weight was again sex-specific: For women only, we found a significant positive correlation between λ_⊥_ and BMI in the anterior part (i.e., in the genu) of the corpus callosum (*p*<0.05, corrected; Figure 4, top row). This result could not be confirmed in men, even when using an uncorrected threshold of *p*<0.05 (Figure 4, bottom row).

**Figure 5:**
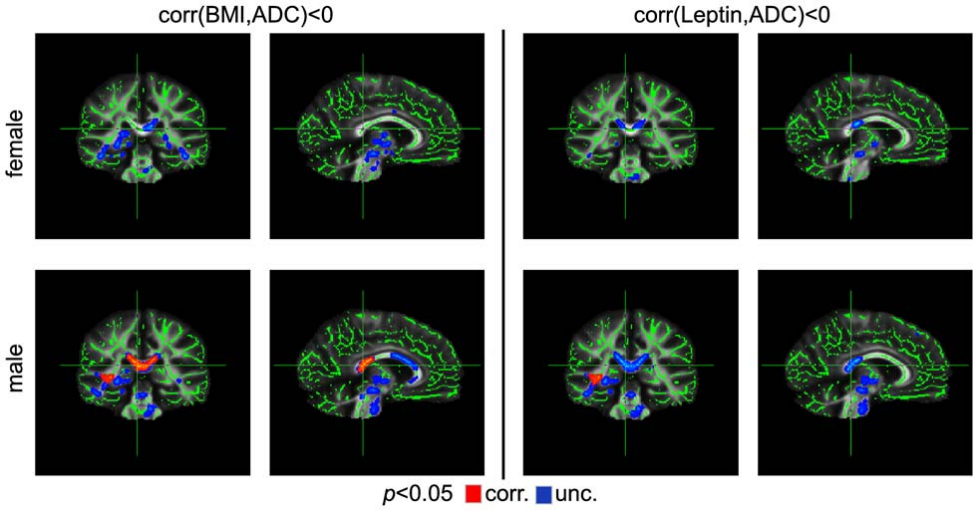
Correlation between ADC and body weight using BMI (left) and leptin levels (right). We found a significant negative correlation between BMI and ADC for men in the splenium of the corpus callosum. Using leptin levels instead of BMI, this correlation was only present without correction for multiple comparisons.

In contrast to the above-mentioned observations, we found a relationship between ADC and BMI only for men, who showed a significant negative correlation in the splenium of the corpus callosum (*p*<0.05, corrected; Figure 5).

Replacing the BMI with the serum leptin level in the statistical analysis did not change any result for the parameters FA, λ_∥_, and λ_⊥_ (Figures 1, 2, 3, 4). This is not surprising given the strong positive correlation between BMI and leptin level in women and men. We found no significant correlation between ADC and leptin levels for women and men using a threshold of *p*<0.05, corrected (Figure 5).

### Region-of-Interest (ROI) Analyses

The ROI analyses were performed for the genu and splenium of the corpus callosum. The results of these parametric analyses reflect the results of the voxel-wise non-parametric permutation tests. Women showed a significant negative correlation between FA and both BMI and leptin (Table 1). A negative correlation between λ_∥_ and BMI was observed for both women and men. However, for the male subjects, the negative correlation between λ_∥_ and leptin was only obtained for the splenium of the corpus callosum. In agreement with the TBSS results, the positive correlation between λ_⊥_ and BMI/leptin was only obtained in women. Finally, the ROI analyses also replicated the negative correlation between ADC and leptin for both women and men in the splenium of the corpus callosum.

**Table 1:** Correlation coefficients (and *p*-values) between diffusion and obesity parameters as obtained from region-of-interest (ROI) analyses in the genu (g) and splenium (s) of the corpus callosum (n.s. = not significant).

		body mass index (BMI)		serum leptin level	
		female	male	female	male
fractional anisotropy (FA)	g	−0.633 (0.002)	n.s. (0.547)	−0.622 (0.003)	n.s. (0.428)
	s	−0.673 (0.001)	n.s. (0.880)	−0.579 (0.006)	n.s. (0.701)
axial diffusivity (λ_∥_)	g	−0.579 (0.005)	−0.538 (0.006)	−0.441 (0.046)	n.s. (0.150)
	s	−0.668 (0.001)	−0.721 (0.000)	−0.692 (0.001)	−0.628 (0.002)
radial diffusivity (λ_⊥_)	g	0.571 (0.005)	n.s. (0.786)	0.623 (0.003)	n.s. (0.814)
	s	0.511 (0.015)	n.s. (0.392)	0.466 (0.033)	n.s. (0.770)
apparent diffusion coefficient (ADC)	g	n.s. (0.484)	n.s. (0.074)	n.s. (0.090)	n.s. (0.409)
	s	n.s. (0.387)	−0.566 (0.003)	−0.459 (0.036)	−0.527 (0.014)

To investigate the potential influence from depression on our findings, we performed correlation analyses between all three BDI values and all four diffusion parameters in both ROIs. No significant correlation between BDI and diffusion parameters were obtained in women or men (*p*>0.05). Partial correlation analyses taking the BDI score into account when investigating the dependence between diffusion and obesity parameters had no marked influence on the results of the correlation analyses. The *r* values changed only marginally when adding the BDI using partial correlation.

### Influence from Instrumental Factors on Diffusion Measurements

It might be argued that the current findings not only reflect brain microstructure but are also influenced by instrumental factors related to body-weight differences including changes in coil loading and loaded *Q*-values between different subjects [54] or vibration artifacts [55]. Extraction of diffusion parameters is known to be prone to errors at low SNR [56]. In particular, anisotropy estimates (e.g., FA) may be progressively biased with decreasing SNR [57]. In our subjects, the average SNR in the corpus callosum decreased from approximately 85 and 101 at a BMI of 20 kg/m^2^ to approximately 75 and 80 at a BMI of 45 kg/m^2^ in the genu and splenium, respectively, which is probably due to variations in loaded *Q*-values.

To address the possibility that the correlations revealed might be influenced by body-weight dependent SNR variations, additional investigations of the correlation between FA and BMI were performed under consideration of the SNR of the non-DW images. Using permutation analyses with the SNR in both genu and splenium as covariates did not change the significant negative correlation between FA and BMI in women. Taking the SNR into account in the ROI analyses, this correlation changed slightly from *r* = −0.633 (*p* = 0.002) in the genu to *r* = −0.612 (*p* = 0.003), and from *r* = −0.673 (*p* = 0.001) to *r* = −0.660 (*p* = 0.001) in the splenium. We also determined the proportion of FA variance explained by SNR variations computing the effect size, η^2^. Differences in SNR only marginally explained the variance of the FA, namely η^2^ = 0.07 (*p* = 0.262) in the genu of the corpus callosum. In contrast, BMI differences across subjects explained a substantial portion of the FA variance in both genu and splenium regardless of whether we added the SNR covariates with η^2^ in the range between 0.35 (*p* = 0.005) and 0.45 (*p* = 0.001). Systematic variations in SNR thus do not adequately explain the observed correlations between BMI and diffusion parameters.

Mechanical vibrations of the patient table stimulated by the low-frequency gradient switching for diffusion-weighting can produce severe artifacts, which may manifest as localized signal loss when there is a strong component of the diffusion-gradient vector in the left–right direction [58]. This signal loss, if misinterpreted as attenuation due to diffusion, produces errors in the quantification of the diffusion tensor and parameters extracted from it. It is currently not known if differences in body weight might lead to a varying degree of attenuation of mechanical vibrations and, hence, to systematic variations of the estimated diffusion tensor components. So far, severe signal loss from vibration effects has been demonstrated only in the parietal lobe in single subjects [58]. A corresponding effect was not observed in our data, which might be due to a different acquisition matrix and use of parallel imaging. Another approach to characterize vibration effects follows the concept for analyzing distortions caused by eddy currents by registering the DW images onto the less-distorted images acquired without diffusion weighting [46]. In particular, there is evidence that vibration-induced motion leads to an affine scaling effect in *x*-direction that can be quantified to obtain a characteristic value for comparing vibration effects in different DTI datasets [47]. For our data, we found no correlation between individual *x*-scaling parameters and any of the diffusion parameters (ADC, FA, λ_∥_, and λ_⊥_). In short, the observed correlations between diffusion parameters and BMI are not adequately explained by vibration effects.

## Discussion

In this study, we systematically investigated the sex-dependence of the relationship between body weight, serum leptin levels and diffusion parameters in WM. Generally, women showed a much stronger correlation between markers of obesity and diffusion parameters than men. The major findings were that greater BMI (or leptin level) was associated with (i) reduced axial diffusivity (λ_∥_) in the entire corpus callosum in both female and male subjects; (ii) increased radial diffusivity (λ_⊥_) in women, predominantly in the genu of the corpus callosum; (iii) reduced FA without a significant ADC change in women, and (iv) decreased ADC predominantly in the splenium of the corpus callosum in men.

Directional water diffusivities derived from the diffusion tensor have been proposed as potential biological markers to detect and differentiate axon and myelin injury. In particular, combined histological and DTI investigations in the mouse optic nerve following retinal ischemia demonstrated that axonal damage initially leads to decreased λ_∥_ but only modest or insignificant changes in λ_⊥_ [33]. By contrast, congenital dysmyelination of shiverer mouse brain increased λ_⊥_ without changing λ_∥_ [59]. In our study, the most widespread alterations were obtained for the putative axon marker λ_∥_, which was consistently reduced throughout the corpus callosum in female and male subjects. Consequently, we may hypothesize that obesity is associated with axonal degeneration. Besides reduced λ_∥_, the putative myelin marker λ_⊥_ increased with increasing BMI in women, predominantly in the genu of the corpus callosum. With regard to our hypothesis above, this would be in line with the assumption that axonal degeneration is paralleled by myelin degeneration. This pattern of changes was previously demonstrated by combined histological and DTI investigations in the mouse model of retinal ischemia [33]. In the current case, longitudinal data were not available, and the assumption of a specific evolution of changes remains speculative.

It has been observed that genetically obese mice show reduced amounts of brain myelin and alterations in the fatty acid composition of myelin in comparison to normal mice of the same strain [60]. From these results, one might expect that myelin degeneration precedes axonal injury. However, the inborn defect in this mouse model already disturbs brain growth during early development when myelination is most rapid. By contrast, there was no evidence for a similar gene defect in our study population, and we may expect normal myelination in early adulthood. It is thus questionable if the genetically obese mouse is a realistic model to predict WM changes associated with elevated BMI in otherwise healthy, young adults.

Further evidence of axonal and myelin degeneration was obtained from a recent study employing proton magnetic resonance spectroscopy (MRS) to investigate the relationship between adiposity and brain metabolites [61]. In particular, lower concentrations of choline-containing compounds with increasing BMI suggesting membrane or myelin alterations were observed in frontal WM. This is consistent with our observation of increased λ_⊥_ predominantly in the genu of the corpus callosum carrying fibers that connect prefrontal areas of both hemispheres. Increased BMI was further associated with reduced concentrations of *N*-acetylaspartate in frontal, parietal, and temporal WM, which indicates axonal dysfunction or loss. Again, this is in line with the widespread observation of decreased λ_∥_ in the entire corpus callosum in our study.

It is interesting to note that the regional pattern of changes in diffusion parameters indicating stronger effects in the anterior sub-regions of the corpus callosum shows similarities with DTI results on aging [62]. As these regions myelinate later and are thought to be more prone to damage during aging, our results may reflect elements of accelerated aging of WM in obese subjects, which has also been proposed on the basis of MRS results [61]. However, such findings could alternatively reflect the adverse effect of elevated BMI on brain development as we cannot rule out the possibility that obese adults were already obese in childhood. Finally, inter-individual differences in brain microstructure might affect gustatory and reward functions in the brain and thereby lead to changes in body weight due to changes in food preferences and intake. We note that the above discussion remains speculative as no longitudinal data are available. Further longitudinal studies are thus advocated to clarify the causalities underlying the observed associations between diffusion parameters, obesity markers, and sex.

Several previous studies revealed differences in WM microstructure in the corpus callosum between male and female subjects. Specifically, men showed significantly larger FA [63], [64], [65], [66] as well as smaller λ_⊥_ values [66]. These findings suggest higher myelination in males, which is also supported by observations of a greater myelin water fraction in the corpus callosum of men [65]. Given such sexual dimorphism, we may speculate that reduced myelination leads to an increased vulnerability of the myelin sheaths in women, which might explain why λ_⊥_ increased significantly with BMI in women but not in men. This is also in line with the observation that the weakest correlations between BMI and diffusion parameters were obtained for the midbody of the corpus callosum (Figures 1, 3, and 4), that is, for the region with the largest diameters of (myelinated) axons [67], [68]. Alternatively, we cannot rule out the possibility that higher myelination inherently reduces the dynamic range for detecting myelin degeneration (i.e., removal of one layer of myelin would correspond to a smaller percentage change in men than in women). Hence, modest demyelination might be less readily detectable by increased λ_⊥_ in males. In this context, it is interesting to note that no sex dependence was observed for metabolic abnormalities associated with BMI [61].

Consistent explanations for the observed alterations in FA and ADC are also achieved from the patterns of changes in the tensor components λ_∥_ and λ_⊥_. Generally, reduced FA may result from increased λ_⊥_ and/or reduced λ_∥_. Both effects (i.e., indications of combined axonal and myelin injury) were simultaneously present in women, yielding a highly significant reduction in FA. By contrast, only λ_∥_ was reduced in men, and the effect on the FA was insufficient to produce a significance change. For the ADC, which is given by ADC = (λ_∥_+2 λ_⊥_)/3 for axially symmetric tensors (as assumed in the context of our study), diminished variations result from opposite signs of changes in the tensor components. Consequently, significant ADC changes were not observed in women but were in men.

The fact that all significant correlations between diffusion parameters and BMI were found in the corpus callosum also raises the question of whether the results are exclusively related to this WM structure. An alternative possibility is that the observed dependencies also exist in wider and less homogeneous WM regions with higher inter-subject variability but may have remained undetected due to a decreased sensitivity of the method in regions with more widespread fiber orientations or crossing tracts. For example, in a study reporting diffusion changes in Wallerian degeneration, Pierpaoli et al. [69] found large changes in diffusion anisotropy only in regions where fibers are arranged in isolated bundles of parallel fibers but not in regions where the degenerated pathway crosses other tracts. A recent study has suggested quantifying different fiber populations within each voxel and relating the corresponding components across subjects [70]. A different approach might use the high angular resolution diffusion imaging data to reconstruct a local fiber orientation distribution function via sharpening spherical deconvolution [71]. In future, this method might give access to the micro-structural properties of different fiber compartments within one voxel in order to compare them across subjects, resulting in a higher sensitivity in areas of crossing fibers. It should be noted that our present results are not limited by crossing fiber populations because the corpus callosum is a bundle consisting of highly parallel fibers.

To shed further light on the close relationship between changes in corpus callosum structure and body weight, longitudinal studies that observe and compare changes in WM structure while losing or gaining weight are necessary. First evidence of a causal relationship between body weight and WM structure was collected by Haltia et al. [17], who reported WM changes as an effect of dieting by analyzing *T*_1_w images using voxel-based morphometry. Other longitudinal studies on GM but not WM provided initial evidence of changing brain structure due to changes in body weight. Increasing body weight appears to be associated with reduced GM volume [72], while weight loss leads to increased GM tissue concentrations [41].

In summary, we show that in female and male subjects with increasing BMI (and serum leptin level), the putative axon marker λ_∥_ was consistently reduced throughout the corpus callosum, suggesting axonal degeneration. Only in women, the putative myelin marker λ_⊥_ significantly increased with increasing BMI (and leptin levels) predominantly in the genu of the corpus callosum, possibly pointing to additional degeneration of myelin. These structural changes were comparable to those reported for the aging brain. This suggests accelerated aging of white matter structure in obese subjects, possibly also affecting gustatory and reward function. Future research should therefore focus on sex-related alterations in brain structure and function due to changes in body weight in order to differentiate between predispositions towards and consequences of obesity.

## Supporting Information

Table S1 List of subjects, with BMI and serum leptin concentrations.
(DOC)
