# Supplementary material for: Sex-Dependent Influences of Obesity on Cerebral White Matter Investigated by Diffusion-Tensor Imaging
Source: PLoS One. 2011 Apr 11;6(4):e18544. doi: 10.1371/journal.pone.0018544 (PMC3073967; doi:10.1371/journal.pone.0018544)
Supplement: Table S1 — List of subjects, with BMI and serum leptin concentrations. (DOC) [file pone.0018544.s001.doc]

| ID | sex | age | weight (kg) | height (m) | BMI (kg/m^2^) | Leptin (ng/ml) |
| --- | --- | --- | --- | --- | --- | --- |
|  |  |  |  |  |  |  |
| A1 | m | 23 | 134 | 1.80 | 41.4 | 23.52 |
| A2 | m | 25 | 75 | 1.75 | 24.5 | <0.20 |
| B3 | f | 31 | 85 | 1.60 | 33.2 | 35.12 |
| BC | m | 41 | 110 | 1.80 | 34.0 | 11.34 |
| BD | f | 22 | 83 | 1.65 | 30.5 | 34.05 |
| BF | m | 31 | 97 | 1.83 | 29.0 |  |
| BN | f | 28 | 109 | 1.74 | 36.0 | 34.15 |
| D4a | f | 23 | 51 | 1.54 | 21.5 | 4.34 |
| D4b | m | 31 | 115 | 1.88 | 32.5 | 8.31 |
| E2 | m | 38 | 150 | 1.72 | 50.7 | 58.65 |
| F5 | m | 30 | 78 | 1.75 | 25.5 | 1.24 |
| H3 | m | 25 | 64 | 1.64 | 23.8 | 2.60 |
| HA | f | 41 | 94 | 1.67 | 33.7 | 47.57 |
| HD | m | 24 | 74 | 1.81 | 22.6 | 4.21 |
| J4 | m | 21 | 105 | 1.83 | 31.4 | 10.96 |
| K8 | m | 22 | 94 | 1.81 | 28.7 | 5.26 |
| K9 | m | 24 | 60 | 1.67 | 21.5 | <0.20 |
| KA | m | 25 | 83 | 1.81 | 25.3 | 3.22 |
| L5 | f | 21 | 93 | 1.74 | 30.7 | 86.17 |
| LG | m | 24 | 120 | 1.86 | 34.7 | 22.03 |
| M1 | f | 29 | 109 | 1.57 | 44.2 | 85.97 |
| M3 | m | 23 | 81 | 1.85 | 23.7 | <0.20 |
| M6 | f | 34 | 54 | 1.69 | 18.9 | 3.60 |
| MB | f | 27 | 88 | 1.76 | 28.4 | 67.19 |
| N2a | f | 23 | 92 | 1.80 | 28.4 | 31.22 |
| N2b | m | 29 | 72 | 1.78 | 22.7 |  |
| P2 | f | 28 | 53 | 1.69 | 18.5 | 5.60 |
| P3 | m | 28 | 110 | 1.83 | 32.8 | 14.22 |
| P8 | m | 35 | 118 | 1.85 | 34.5 | 14.98 |
| P9 | m | 27 | 105 | 1.76 | 33.9 |  |
| R1 | m | 26 | 82 | 1.85 | 24.0 | 3.53 |
| R3 | m | 32 | 90 | 1.90 | 24.9 |  |
| R5 | f | 22 | 74 | 1.78 | 23.4 | 10.97 |
| R9 | f | 22 | 120 | 1.73 | 40.1 | 87.59 |
| RB | f | 19 | 82 | 1.77 | 26.2 | 44.23 |
| S1 | f | 26 | 82.5 | 1.68 | 29.2 | 20.93 |
| S5 | f | 26 | 52 | 1.63 | 19.6 | 4.36 |
| S9 | m | 25 | 81 | 1.83 | 24.2 | 0.35 |
| SG | f | 20 | 77 | 1.60 | 30.1 | 58.83 |
| SH | m | 24 | 86 | 1.86 | 24.9 | 0.73 |
| SJa | f | 24 | 128 | 1.68 | 45.4 | 46.00 |
| SJb | f | 25 | 90 | 1.72 | 30.4 | 13.16 |
| SL | m | 24 | 83 | 1.89 | 23.2 | 1.60 |
| SO | f | 26 | 73 | 1.63 | 27.5 | 31.48 |
| T5 | m | 24 | 73 | 1.75 | 23.8 | <0.20 |
| T6 | f | 28 | 80.5 | 1.68 | 28.5 | 13.47 |
| V1 | m | 24 | 89 | 1.73 | 29.7 | 5.30 |
| W9 | f | 20 | 62 | 1.69 | 21.7 | 12.16 |
| WB | f | 22 | 91 | 1.65 | 33.4 |  |
